# A Further Contribution to the Therapeutics of Heroin

**Published:** 1899-05

**Authors:** Julius Weiss

**Affiliations:** Vienna


### A FURTHER CONTRIBUTION TO THE THERAPEUTICS OF HEROIN.

By JULIUS WEISS, M. D., Vienna.

SIMULTANEOUSLY with the publication of my experience in the October number of *Die Heilkunde* appeared an article by Floret in the *Therapeutische Monatshefte*, No. 9, detailing his results with heroin. Floret likewise came to the conclusion that this remedy is an excellent sedative for coughs, both in cases of acute as well as chronic bronchitis, since it not only alleviates the subjective symptoms and the pains in the chest, but also greatly diminishes the desire to cough. In twenty-five cases of pulmonary tuberculosis the drug afforded much relief in doses of 0.005 to 0.01 gm. Strube, *Berliner Klinische Wochenschrift*, No. 7, 1898, tried heroin in the Medical University Clinic of Professor Gerhardt, of Berlin, and experiments made by him on animals showed that heroin has a particularly strong influence upon the respiration, which is manifested by doses too small to produce any narcotic effects. He administered pills of 0.005 gm. and in cases where this dose proved ineffective, increased it to 0.01 and 0.015 gm. In every instance in which Strube made use of heroin, it exerted a perceptible influence upon the breathing, diminishing the frequency of respiration and the tussal irritation. Altogether he employed it in fifty cases, comprising chiefly phthisical subjects.

Heroin in the pure state has the disadvantage of being insoluble in water, so as to limit its administration to pills and powders. For this reason the preparation of a combination of heroin soluble in water, the so-called heroin hydrochloric, should be regarded as a material advantage. I have prescribed heroin hydrochloric in twentyfive cases and found it as efficient as the heroinum purum. I administered it dissolved in aqu. amygdal. amar; in solutions in distilled water with addition of mixt. gummos. In a few cases I found a combination with creosote, salipyrin and tincture of strophanthus useful.

My clinical material consisted of patients suffering from phthisis, simple primary bronchitis and bronchitis secondary to valvular diseases of the heart. As is well known, the latter class of cases is often very distressing and difficult to relieve, especially if it is impossible to establish adequate cardiac compensation.

Our customary cardiac remedies are entirely inefficient in certain cases, especially lesions of the aortic valves, arterial sclerosis without valvular trouble, and also in certain affections of the cardiac muscle, while in those of the mitral valve and in another group of diseases of the cardiac muscle they exert a prompt influence. If we succeed in establishing compensation, then the secondary bronchical catarrh will vanish. This fact must be especially emphasised as, unfortunately, morphine preparations are sometimes prescribed for patients for whom cardiac remedies would prove more serviceable.

The employment of morphine and its substitutes is, however, especially indicated in those affections of the heart in which cardiac remedies are ineffective, or contra-indicated. In this very class of cases, heroin appears to me to present a special advantage over morphine or codeine since even in small doses it deepens and prolongs the respiration and simultaneously exerts a sedative influence, without any decided narcotic effect, at least in the small doses required. In two cases in which distinct signs of angina pectoris with arterial sclerosis were visible, heroin greatly alleviated the stenocardiac disturbances. In four cases of influenza in which the pulmonary symptoms presented the picture of bronchitis sicca, heroin produced a marked improvement in the disagreeable pains in the chest and in the bronchial irritation.

One of the chief advantages of this new substitute of morphine seems to be that it manifests so decided an effect in comparatively so small doses, that it may be given without risk even in those cases in which the heart and lungs are conjointly affected. The advantage of the soluble heroin hydrochloric over heroin itself consists in that it is often advisable to prescribe the drug in solutions and mixtures in preference to powders and pills, not only because some patients object to the latter form of administration, but also because the first form enables us to give heroin in combination with other efficient medicaments.—*Die Heilkunde*, February, 1899.

